# Etmopteridae bioluminescence: dorsal pattern specificity and aposematic use

**DOI:** 10.1186/s40851-019-0126-2

**Published:** 2019-03-06

**Authors:** Laurent Duchatelet, Nicolas Pinte, Taketeru Tomita, Keiichi Sato, Jérôme Mallefet

**Affiliations:** 1 Marine Biology Laboratory, Earth and Life Institute, Catholic University of Louvain, Place Croix du Sud 3, 1348 Louvain-la-Neuve, Belgium; 2 Okinawa Churaumi Aquarium, 424 Ishikawa, Motobu-cho, Okinawa prefecture 905-0206 Japan; 3 Zoological Laboratory, Okinawa Churashima Research Center, 888 Ishikawa, Motobu-cho, Okinawa 905-0206 Japan

**Keywords:** Etmopteridae, Dorsal pattern, Bioluminescence, Intraspecific recognition, Aposematism, Spines

## Abstract

**Background:**

In the darkness of the ocean, an impressive number of taxa have evolved the capability to emit light. Many mesopelagic organisms emit a dim ventral glow that matches with the residual environmental light in order to camouflage themselves (counterillumination function). Sharks use their luminescence mainly for this purpose. Specific lateral marks have been observed in Etmopteridae sharks (one of the two known luminous shark families) suggesting an inter/intraspecific recognition. Conversely, dorsal luminescence patterns are rare within these deep-sea organisms.

**Results:**

Here we report evidence that *Etmopterus spinax, Etmopterus molleri* and *Etmopterus splendidus* have dorsal luminescence patterns. These dorsal patterns consist of specific lines of luminous organs, called photophores, on the rostrum, dorsal area and at periphery of the spine. This dorsal light seems to be in contrast with the counterilluminating role of ventral photophores. However, skin photophores surrounding the defensive dorsal spines show a precise pattern supporting an aposematism function for this bioluminescence. Using in situ imaging, morphological and histological analysis, we reconstructed the dorsal light emission pattern on these species, with an emphasis on the photogenic skin associated with the spine. Analyses of video footage validated, for the first time, the defensive function of the dorsal spines. Finally, we did not find evidence that Etmopteridae possess venomous spine-associated glands, present in Squalidae and Heterondontidae, via MRI and CT scans.

**Conclusion:**

This work highlights for the first time a species-specific luminous dorsal pattern in three deep-sea lanternsharks. We suggest an aposematic use of luminescence to reveal the presence of the dorsal spines. Despite the absence of venom apparatus, the defensive use of spines is documented for the first time in situ by video recordings.

**Electronic supplementary material:**

The online version of this article (10.1186/s40851-019-0126-2) contains supplementary material, which is available to authorized users.

## Background

Deep in the ocean, a great many taxa have evolved the capability to emit light [1, 2]. This phenomenon, called bioluminescence, is a mechanism whereby organisms emit visible light by biochemical reactions [1, 3, 4]. Functions of bioluminescence are mainly divided into three categories: predation, avoid predation (interspecific) and intraspecific communication [1, 3–6]. Among these organisms, sharks are the first vertebrate to utilize this phenomenon [1, 7, 8]. Currently, there are two families of luminous deep-sea sharks (Etmopteridae and Dalatidae) which are capable of emitting a blue-green light (from 460 to 486 nm according to the species) thanks to thousands of tiny luminous organs, called photophores, mainly present on the ventral skin epidermis [7, 9–11]. The photophore structure, conserved in the genus *Etmopterus*, is composed of a “deep” pigmented sheet of embedded cells responsible for the light emission, called photocytes, surmounted by an iris-like structure topped externally by one or two lens cells [12–14]. Shark luminescence has been suggested to have several ecological roles. Firstly, like a large number of mesopelagic organisms emitting a continuous ventral glow similar to the down-welling light, lanternsharks use their ventral light to disrupt their silhouette and avoid being seen by predators swimming below; this is the counterillumination mechanism [1, 6, 10, 15]. Secondly, interspecific and intraspecific communication have been suggested: (i) the presence of species-specific lateral flank marks may provide a way to facilitate reproductive isolation hence the high species richness in *Etmopterus* genus [8, 16], while (ii) sexual dimorphism is observed in the Etmopteridae species [12]. Aposematism is also suggested, provided by the spine associated photophore luminescence [17].

Previous studies have demonstrated that shark spines fulfill numerous functions, these include improving hydrodynamics of the organism and serving as a mechanism for defense. The presence of venomous glands associated with the posterior side of the spine in Heterodontidae and Squalidae are evidence for this function [18–22]. However, there is now in situ evidence for a defensive function of the dorsal spine in Etmopteridae. In contrast to ventral luminescence, dorsal luminescence is rare in the ocean and has received less interest, probably because it is easily detectable in contrast to the darker background from the deeper waters underneath the organism. This dorsal pattern is usually utilized for predation [1, 5], as indicated by the dorsal lure located above the jaws in anglerfish species [23] or for an anti-predatory function, where the light acts as an aposematic warning signal [24–26].

In this study, we investigated dorsal light emission in three deep-sea shark species from the Etmopteridae family: the velvet belly lanternshark, *Etmopterus spinax* (Linnaeus, 1758); the slendertail lanternshark, *Etmopterus molleri* (Whitley, 1939); and the splendid shark, *Etmopterus splendidus* (Yano, 1988). These three species are small deep-sea sharks (see Table 1) occurring at depths ranging from 200 to 500 m, where sunlight dimly penetrates the water column [27–29]. Due to their small sizes, these sharks are preyed upon by larger organisms, such as other elasmobranch species including *Dalatias licha*, *Echinorhinus cookei* and large Hexanchiformes species ([30–33], JM personal communication). Lanternsharks are characterized by mineralized spines in front of dorsal fins, a subterminal notch on the dorsal part of the caudal fin and a specific pattern of light organs on the ventral and lateral body surface [11–13, 17]. A recent study highlighted the specialization of the visual system in luminous deep sea sharks compared to non-luminous species: (i) longer rod outer segments, higher rod densities and larger eye:body size ratio, these are in favor of high light sensitivity; (ii) maximal absorption wavelengths in visual pigments (484–491 nm) for perception of bioluminescent emissions (476 nm *E. splendidus*, 486 nm *E. spinax* and 488 nm *E. molleri*) and downwelling light; (iii) the presence of a secondary dorsal arch with a high density of rods in the retina which facilitates the detection of moving objects in the inferior visual field [34].

**Table 1:** Mean morphological values. N: number of specimens; ♀: female; ♂: male

Species	N	Total length (cm)	Fork length (cm)	Pre-caudal length (cm)	Weight (g)
*Etmopterus spinax*	25 ♀	46.3 ± 1.1	39.6 ± 1.1	35.6 ± 1.1	416.1 ± 23.8
	6 ♂	40.0 ± 1.3	35.2 ± 1.3	31.3 ± 1.3	248.1 ± 34.2
*Etmopterus molleri*	15 ♀	42.7 ± 2.1	36.7 ± 1.9	33.9 ± 1.4	217.6 ± 43.1
	9 ♂	40.1 ± 0.9	33.8 ± 0.9	31.6 ± 0.7	165.6 ± 17.2
*Etmopterus splendidus*	3 ♂	21.9 ± 1.6	19.6 ± 1.2	17.5 ± 0.9	/

Our results reveal the presence of a dim blue-green light from the dorsal epidermis for these sharks. We report a species-specific dorsal pattern of photophores that may be utilized for species recognition, schooling, and other intraspecific communication. We also provide details of the specific luminous pattern associated with the spine in different Etmopteridae species. In this study, the first evidence of dorsal spine in a defensive use, is recorded by in situ video footages of Etmopteridae sharks. While CT and MRI scan images indicate that Etmopteridae sharks do not show the presence of a venomous gland associated with their spines.

## Materials and methods

### Etmopteridae sampling

The three elasmobranch species come from two different regions. *E. spinax*, is mainly found in the north-east part of the Atlantic, and were collected in the Raunefjord (60°15′54″ N; 05°07′46″ E) next to Bergen in Norway during winter 2017. A total of 31 specimens were sampled during this field session. They were caught using a deep-long line at a depth ranging from 180 to 250 m. Specimens were transferred in a dark cold tank (4 °C) and brought to the Espegrend marine field station where they are kept alive in a seawater tank placed in a cold dark room (4 °C) until manipulations.

*E. molleri* and *E. splendidus* were collected in the East China Sea (26°28′94″ N; 127°41′20″ E) near the coast of Okinawa Island (Japan). They were fished using a bottom hook-and-line method at a depth ranging from 480 to 510 m. Data on *E. splendidus* and *E. molleri* were collected during the fishing seasons winter 2011 and winter 2016, respectively. Three specimens of *E. splendidus* and 24 specimens of *E. molleri* were collected. All specimens were transferred to oxygen saturated plastic bags filled with seawater and transferred in a refrigerated box to the Okinawa Churaumi Aquarium where they were kept alive in a cold dark tank filled with seawater (13 °C) until manipulations.

Data on the collected specimens are summarized in Table 1.

### Dorsal luminescence pattern analysis

Dorsal photos of luminous and living shark were taken with a Sony alpha 7S II camera (Sony Corporation, Japan), these images were analyzed, digital noise was removed using Photoshop software (Adobe; San Jose, CA, USA). Close-up images of the photogenic structure associated with the dorsal spines was also completed with the same software.

### Spine-associated luminous structure analysis

Since photophores located on the dorsal fin (SAP) highlighting the spine were documented in *E. spinax*, we analyzed the structure and orientation of photophores located around the spines in different Etmopteridae species with the aim to compare arrangements among species.

Captive sharks were euthanized by a knock on the chondrocranium followed by an incision at the level of the spinal cord. The local rules for experimental fish care and the European regulation for research animal handling were followed. Shark dorsal skin, spine and fin were dissected and directly stored in 4% paraformaldehyde phosphate buffer saline (PBS) for 12 h at 4 °C, and stored in PBS until further use. A 1.5 cm diameter skin patch around the spine was removed and separated from the spine. For histological analyses, dorsal skin, fin epidermis and skin patches were bath in PBS with increasing sucrose concentrations (10% for 1 h, 20% for 1 h and finally, overnight in 30% sucrose), embedded in O.C.T. compound (Tissue-Tek, The Netherlands) and finally, rapidly frozen at − 80 °C. Thin sections (10 μm) were cut with CM3050 S. Leica cryostat microtome (Germany) and were laid on Superfrost-coated slides (Thermo Scientific, Waltham, MA, USA) and left overnight to dry. Slides were analyzed using an epifluorescence microscope and a light microscope (Leitz Diaplan, Germany) equipped with a Nikon Coolpix 950 camera (Nikon, Japan). General morphology of the photophore, distance and the inclination angle (α) measured in relation to the spine location were all described. The photophore inclination angle (light pathway) was evaluated by taking the difference between the perpendicular to the iris opening as the reference axis and the line passing through the central point of the largest lens. Photophore distance was measured from the center of the light organ to the base of the spine. These two measurements were taken on pictures via ImageJ software [35]. Statistical analyses were performed with JMP® software (JMP®, Version 13. SAS Institute Inc., Cary, NC, 1989–2007.). The Gaussian distribution respected an ANOVA followed by a Tukey-test to reveal significant differences.

### Computed tomography and MRI analyses

Knowing that spine associated venom glands were detected as soft tissue located at the posterior side of the spine [18–20, 22], magnetic resonance imaging (MRI) data of the *E. spinax* spine and the associated tissues were obtained thanks to a Bruker Biospec 11,7 T (Bruker BioSpin, Ettlingen, Germany) in order to visualized the presence/absence of a specific venomous structure. A bird-cage coil with an internal diameter of 40 mm was used in emission/reception mode. The run sequence was Flash type with the following parameters: TE: 3.2 ms; TR: 320 ms; FA: 25°; matrix size: 396 × 396; field of vision of 30 × 30 mm^2^; ten non-continuous slides separated from 350 μm (center to center); resolution: 76 × 76 × 250 μm^3^; number of repetitions: 700. Computed tomography (CT) data of *E. spinax* were acquired using a cone beam micro-CT scanner (NanoSPECT/CT, Bioscan inc., Washington D.C., USA) with the following characteristics: spatial resolution: 48 μm; X-ray tube voltage: 45 kVp; number of projections: 360; exposure time: 1000 ms. The CT projections were reconstructed with a voxel size of 0.111 × 0.111 × 0.11 mm^3^ by ray-tracing based filtered back projection.

### Video footage

During the field session in November 2016, video footage of *E. molleri* was collected at one location in the oriental China Sea (26°34′94″ N; 127°45′20″ E). Two deployments occurred at depths of 500 and 540 m. Each video device was on the seabed during a period of 2 h. The underwater video system was designed by ourselves. Video footage was taken by a GoPro Hero 4 (GoPro, Inc., San Mateo, CA, USA) placed in a special underwater case, benthic 2 (Group B Distribution Inc., Jensen Beach, FL, USA) and fixed on the metal frame. The bait consisted of 1 kg of cephalopod and mackerel in a metal cage fixed to the frame by a steel bar. Lighting was provided by LED light in a housing, GPH-1750 M (Group B Distribution Inc.) and provided us a clear view until four meters and did not appear to disturb shark behaviors. The depth was recording using the sonar system of the boat.

## Results

### Dorsal luminescence pattern

**Fig. 1:**
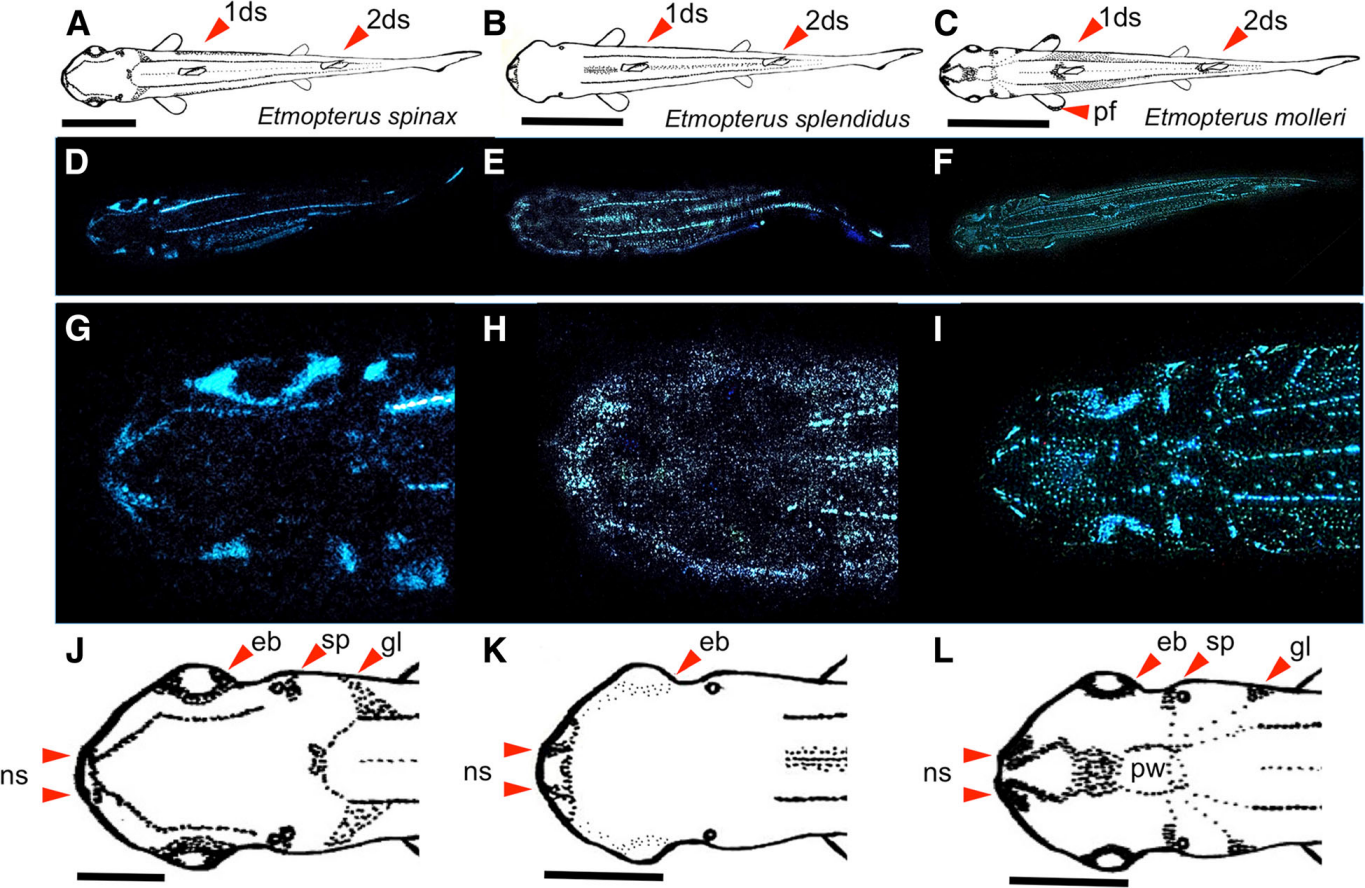
Dorsal luminescent patterns of *E. spinax, E. splendidus* and *E. molleri*. Schematic view of the dorsal patterns; images of dorsal luminous patterns and head close-up; schematic representation of the dorsal head luminous patterns of *E. spinax* (**a**, **d**, **g**, **j**), *E. splendidus* (**b**, **e**, **h**, **k**) and *E. molleri* (**c**, **f**, **i**, **l**) respectively. 1ds: first dorsal; 2ds: second dorsal; eb: eyeball; gl: gills; ns: nostril; pf: pectoral fin; pw: pineal window; sp.: spiracle. Scale bars 8 cm (**a**, **b**, **c**) and 2 cm for (**j**, **k**, **l**)

Analysis of different dorsal luminous pictures allowed us to build schematic drawings of the dorsal side of each species (Fig. 1). Analyses of these patterns for each species revealed some luminous arrangements common to all species and others species-specific patterns: (i) the three luminous longitudinal lines from the back of the head to the beginning of the caudal fin were common for the three species studied (Fig. 1a-f), even if the median line of *E. splendidus* was denser than median line of the two other species; (ii) a relatively brighter luminous mark located on the ventral side of the caudal fin dorsal lobe was observed; (iii) on the rostrum dorsal side, despite many different patterns, the photophore aggregation around nostrils was a common feature (Fig. 1g-l); (iv) a brighter luminous spot appeared next to the dorsal spines in these three species.

In addition to these similarities, many different luminous arrangements occurred between these three species, mainly focused on the rostrum and pectoral fins. Indeed, we saw that *E. spinax* and *E. molleri* show aggregation of the luminous organs around the eyes, next to spiracles and at the edge of gill slits. A luminous impala horn shape pattern was observed on the *E. spinax* head (Fig. 1g, j) and a particularly luminous shape arrangement was visible on *E. molleri* head (Fig. 1i, l). The pineal window surrounded by a circle of luminous dots from which radiating lines connecting spiracle, gills slits, and dorsal lines were visible. Between this window and the nostril, a luminous V shape and blotch were observed on the rostrum. Moreover, *E. molleri* shows a specific luminous zone on pectoral fins (Fig. 1c, f).

In contrast to these two species, *E. splendidus* possessed no specific luminous zones on the rostrum (Fig. 1h, k).

The dorsal light emission is about one order of magnitude dimmer than ventral light emission. We did not distinguish any sexual differences during our observations, although we observed that transferring the shark from a captivity tank to an aquarium induces a transient increase of bioluminescence lasting around 10 min.

### Spine-associated luminous structure

**Fig. 2:**
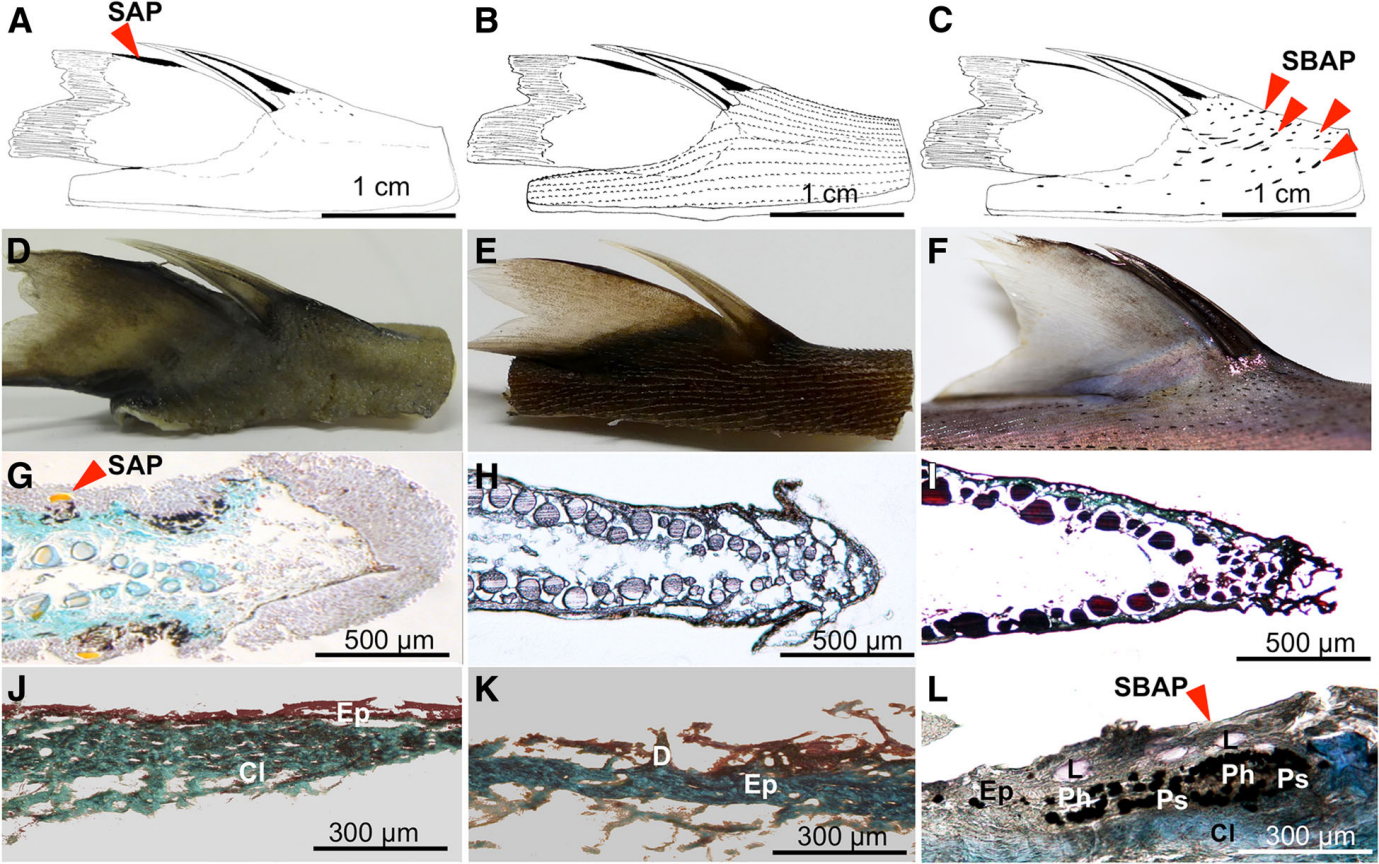
Spine-associated luminous structure analyses. Schematic view of the second dorsal spine; second dorsal spine images; second dorsal fin cross sections and skin base of the second spine of *E. spinax* (**a**, **d**, **g**, **j**), *E. splendidus* (**b**, **e**, **h**, **k**) and *E. molleri* (**c**, **f**, **i**, **l**) respectively. All sections are Masson’s trichrome stained. Cl: connective layer; D: dermal denticle; Ep: epidermis; L: lens; Ph: photocytes; Ps: pigmented sheath; SAP: spine associated photophores; SBAP: spine base associated photophores

**Fig. 3:**
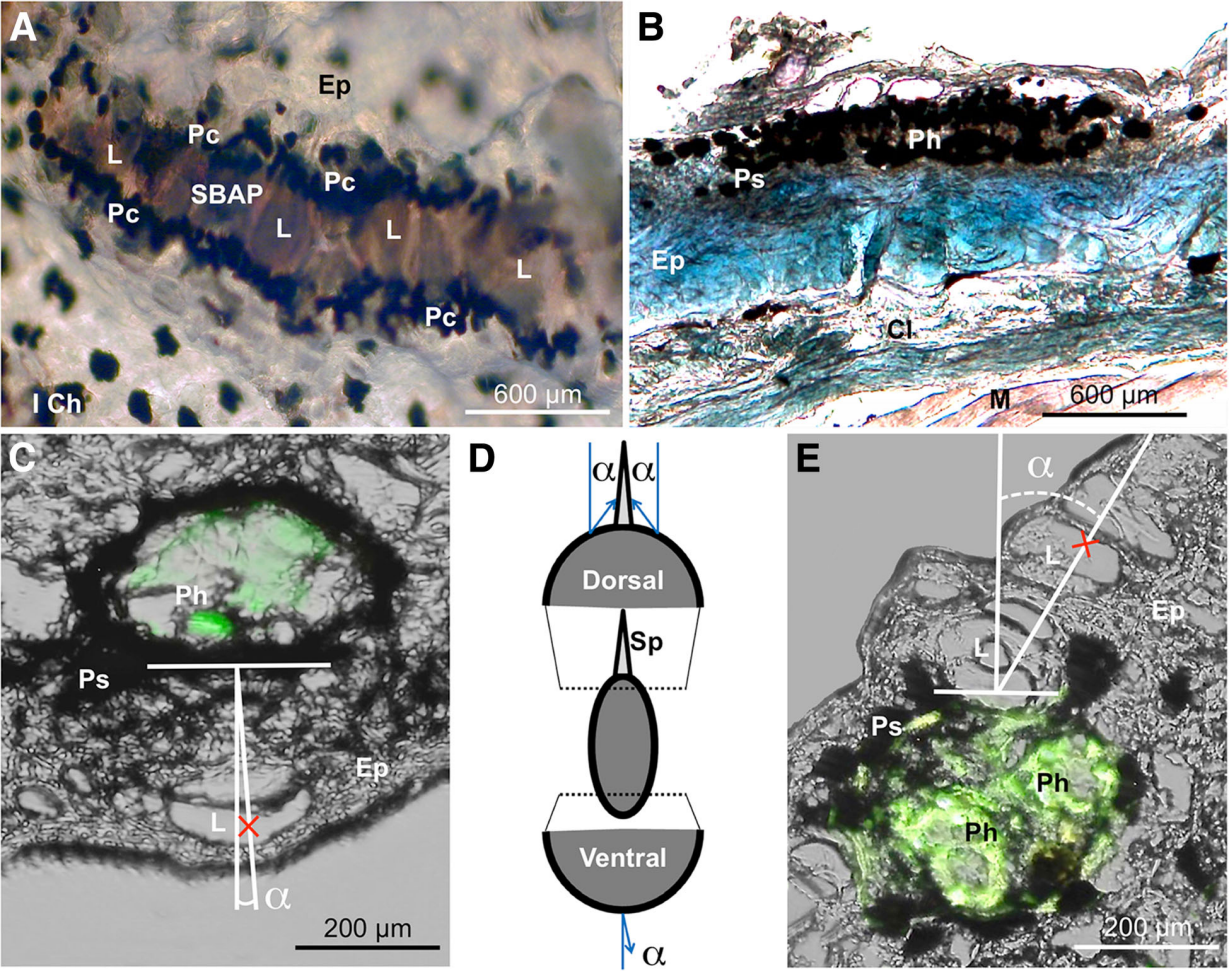
Spine base associated photophore (SBAP) close up structure. **a** External view of SBAP showing lens cells and the pigmented crown. **b** Transversal section of SBAP showing the internal structure of this new photophore type. **c** Longitudinal section of ventral photophore with a mean inclination angle < 3°. **d** Schematic shark illustration showing ventral and SBAP inclination angles (**e**) Longitudinal section of SBAP with a mean inclination angle ±35° turned toward the spine. Red crosses correspond to the center of the biggest lens cell; ɑ: angle; Ep: epidermis; I Ch: isolated chromatophore; Cl: connective layer; L: lens; M: muscle; Pc: pigmented crown; Ph: photocytes; Ps: pigmented sheath; Sp: spine

**Fig. 4:**
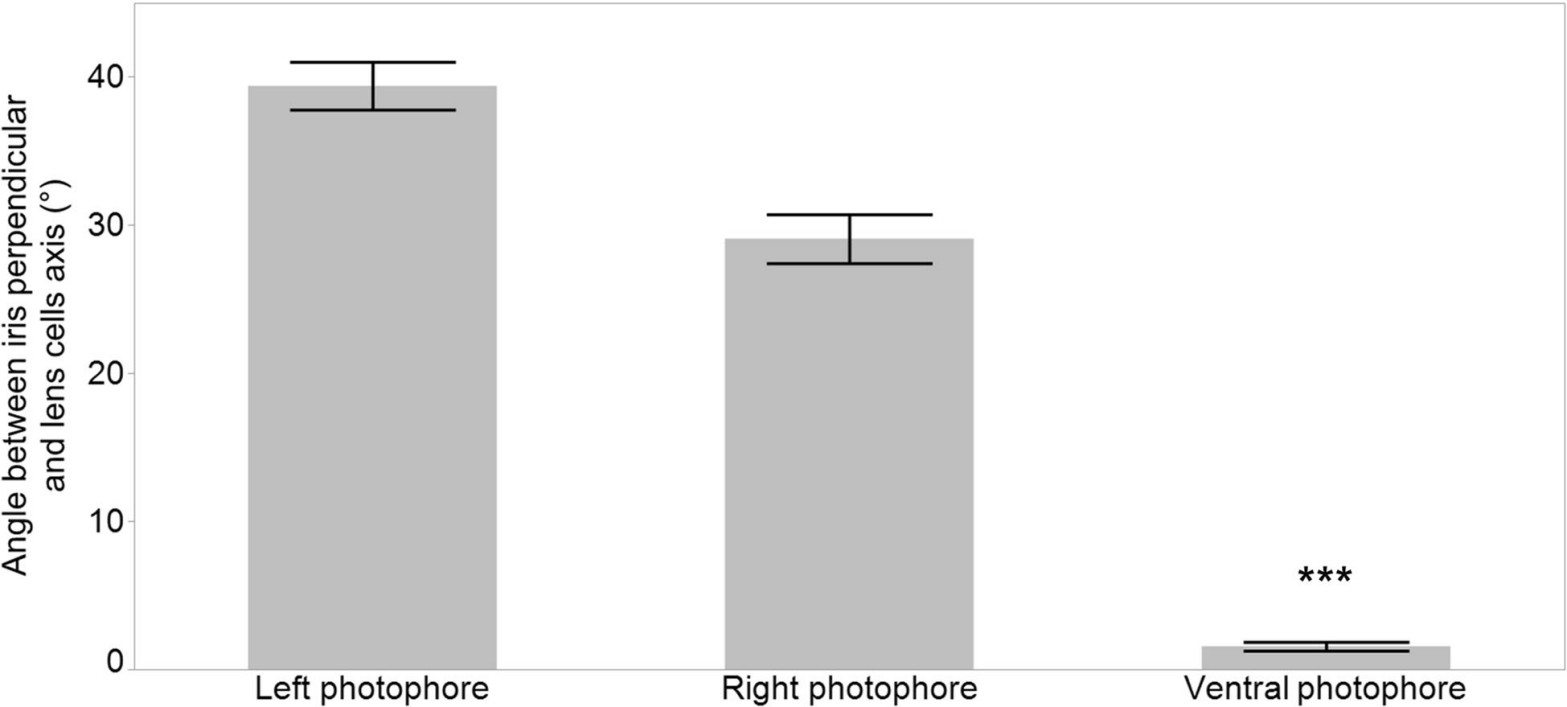
Mean inclination angle between iris perpendicular and the lens cells axis of left and right spine base associated photophores (SBAPs) and ventral photophores

Close-up observations of the dorsal fin body region were performed to analyze spine-associated luminous structure for the three studied species (Fig. 2). Dissected spines with the 1.5 cm skin-associated allowed us to characterize two different spine-associated photogenic cluster patterns. Claes et al., (2013) describes the patterns in *E. spinax*, which consists of spine associated photophore, called SAP. These photophores are precisely localized along the fin anterior ridge facing the spine (Fig. 2a, d). This pattern was found and can be visualized through the thin dark strip on the antero-dorsal part of the fin (Fig. 2a, d). At this location, the photophores are oriented directly towards the spine and illuminate it (Fig. 2g). *E. molleri* did not show any photophores on the dorsal fin ridge (Fig. 2c, f, i) but careful examination of dorsal skin revealed the presence of luminous structures on the front and on each side of the spine base, these photogenic structures were named spine base associated photophore (SBAP) (Fig. 2c, l). These photophores group together in small lines all around the spine with a rostro-caudal orientation (Fig. 2c, f). The size of SBAP clusters in front of the spine measured 775 μm ± 322 and side clusters were estimated as 443 μm ± 543 (Fig. 3a). Longitudinal and transversal sections across SBAP allowed us to describe this new photophore type (Fig. 3b, e). SBAP is mainly composed of numerous photocytes forming an antero-posterior elongated tube shape, surrounded by pigmented cells and surmounted by at least five lens cells (Fig. 3a, b, e). These SBAPs are localized at a mean distance of 4.75 ± 0.74 mm from the spine base (*n* = 210 SBAP observed). To find out if these photophore lines were able to illuminate the spines, the inclination angles (α) between the SBAP (Fig.3d, e) and ventral photophores (Fig. 3c, d) were measured. Ventral photophores showed an angle of 1.6° ± 0.8 (*n* = 42) significantly different from right side SBAP presenting a value of 29.1° ± 7.2 (*n* = 36) and left side, a value of 39.4° ± 9.1 (*n* = 50) (Fig. 4; Tukey *p* < .001). Regarding *E. splendidus,* no specific spine associated photophores (SAP/SBAP) could be highlighted neither on the dorsal fin nor at the level of the spine base skin (Fig. 2b, e, h, k).

### Spine structure and associated putative gland

**Fig. 5:**
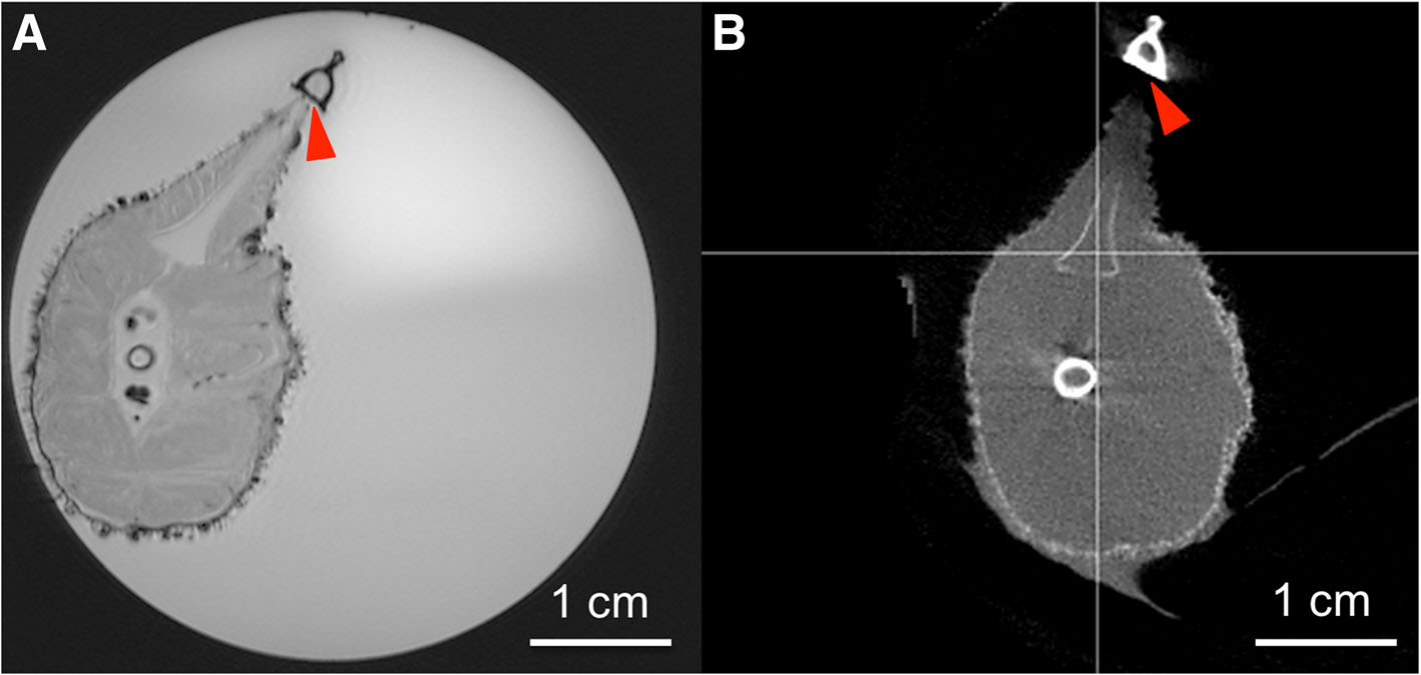
Spine structure. Spine structure analyses by MRI image showing any evidence of soft tissue at the posterior side of the spine (red arrowhead) (**a**). Internal spine structure analyses by micro CT scan showing any evidence of a duct/canal at the posterior side of the spine hard tissue (calcified structure), red arrowhead showing the calcified tissue at the posterior side of the spine without any duct/canal (**b**). In both analyses, the only hollow on the spine, at the center, is filled with cartilaginous matrix

To visualize a putative venom apparatus in Etmopteridae, MRI analysis was performed on fixed specimens; image analyses do not show any evidence of a soft tissue between the posterior side of the spine and the dorsal fin (Fig. 5a; Additional file 1). CT scan analyses did not allow to highlight any canal/duct on the anterior side of the spine that can be used for venom injection into a potential predator (Fig. 5b; Additional file 2).

### Video footage

**Fig. 6:**
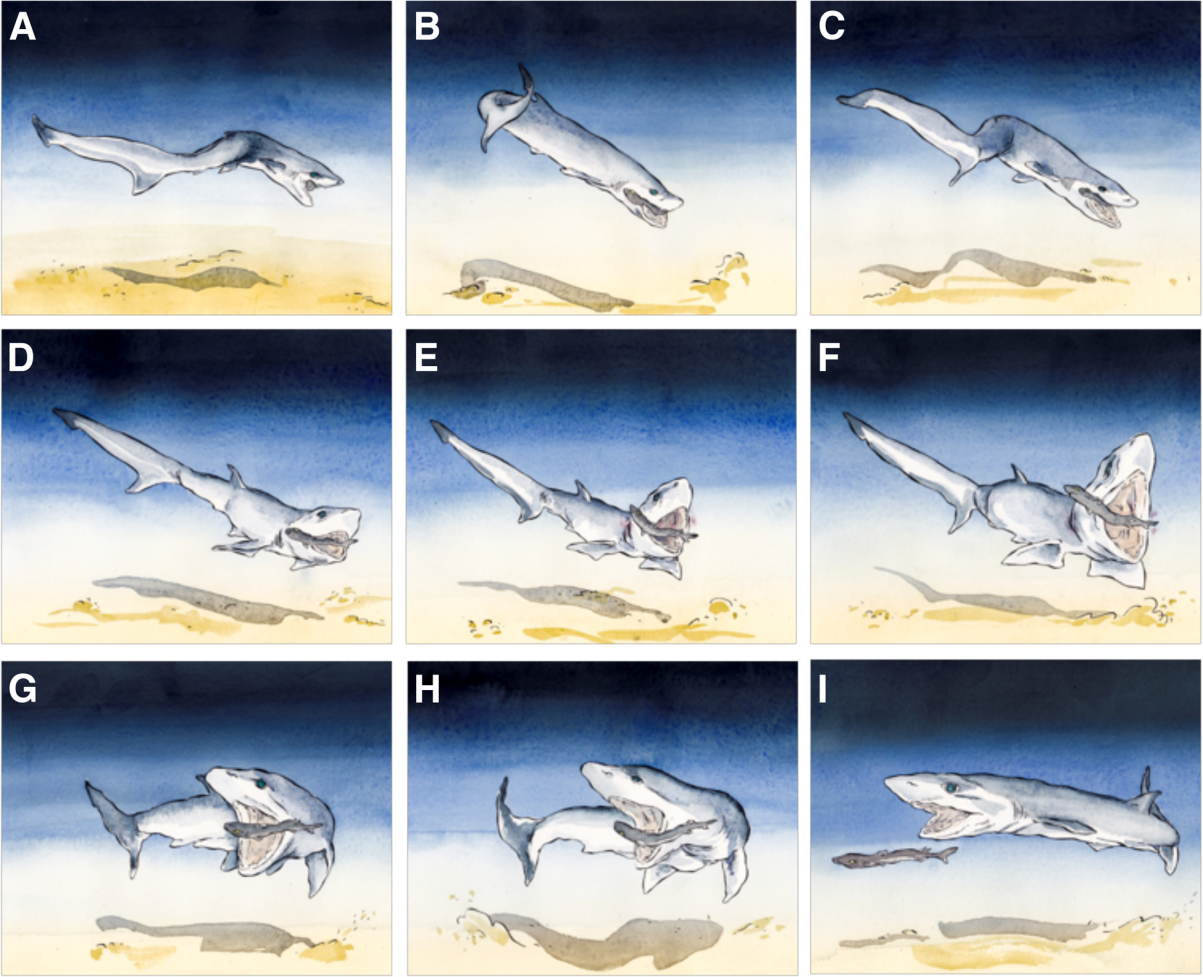
Schematic time-lapse of hunting behavior of *H. perlo* on an *E. splendidus* where the spine defensive function is illustrated (images taken from video footage see supplemental materials)


The video footage filmed in Okinawa allowed us to highlight the defensive function of the dorsal spine in Etmopteridae (Fig. 6). The video shows a sharpnose sevengill shark, *Heptanchrias perlo* (Bonnaterre, 1788), catching an *Etmopterus splendidus* (Fig. 6a), attempting to bite it twice (Fig. 6b, c, d), the shark then opened its jaws widely (Fig. 6e, f, g) letting the *Etmopterus* escape (Fig. 6h, i). The original recording is provided as Additional file 3.

**Additional file 3:** Video recording of *Heptanchrias perlo* attack on an *Etmopterus splendidus* body. (MOV 6040 kb)


We also observed sequences where a sevengill shark attempts to catch a lanternshark by the tail avoiding the spine sting, as shown on the Additional file 4. During field collections, caught lanternsharks were observed on the hook with the tail cut off or the belly showing bite signs (Additional file 5).


**Additional file 4:** Video recording of *Heptanchrias perlo* attack on an *Etmopterus molleri* tail. (MOV 2448 kb)


## Discussion

Among luminous organisms of the mesopelagic zone, dorsal luminescence is rare, as it seems counter-intuitive to produce a dorsal luminous signal making the emitter highly visible against the darkness of the deeper waters, while most organisms produce a ventral light to counterilluminate and escape from the sight of predators [6, 36–38]. We observed specific rostrum and dorsal light patterns, which may be utilized for intraspecific communication (schooling, mating), similarly to the dorsal caudal gland of certain Myctophidae fishes [39]. Assuming this intraspecific function, we suggest that this dorsal luminous pattern, like the specific flank marks of Etmopteridae, may have contributed to the large evolutionary radiation and speciation occurring in the *Etmopterus* genus [8, 16]. The dorsal lines already described in daylight by taxonomists were never referred as bioluminescent lines [40–42]. The species-specific patterns could be a useful feature for the Etmopteridae species determination/taxonomy, and therefore used as a new morphological phylogeny criterion.

However, Claes et al. (2013) have suggested the dorsal aposematic function of light for *E. spinax,* where the light from SAP (spine associated photophore) is strongly transmitted by the dorsal spine making it visible for potential predators. Our results show that handling the lanternshark species induces an increase of bioluminescence and the presence of a brighter spot of luminescence surrounding the spine areas, these findings agree with this aposematic function. However, in *E. molleri* we found a new elongated photophore type with numerous lenses whose orientation points towards the dorsal spine, we call these spine base associated photophore or SBAP. These are much larger than the photophore commonly described for Etmopteridae [7, 14, 43], and may represent a cluster of numerous photophores aiming to light up the spine. Consistent with the Squaliformes phylogeny, our results reveal a morphologically divergent evolution of photophore (SAP/SBAP) within Etmopteridae in order to reach a convergent functionality, aposematism. The primary homology hypothesis seems unlikely due to the positioning of the three studied species [44]. The use of conspicuous signals to warn predators of unprofitability, aposematism [45–48], has been suggested for bioluminescent organisms in terrestrial and oceanic environments [17, 24–26, 49–51].

The presence of a venomous gland at the spine base in two shark families (Squalidae and Heterodontidae) was considered proof of a defensive function of this spine [18–20, 22]. Despite that no evidence of such gland was shown by MRI and CT scan analysis at the level of the dorsal spines in Etmopteridae, our video recordings and images are the first in situ validation of a defensive use of the dorsal spines. Attacks by predators at the level of the belly and tail of Etmopteridae seem to indicate that learning behavior has led predators to specifically avoid dorsal spines during predation attempts.

## Conclusion

This work highlights for the first time a species-specific luminous dorsal pattern in three deep-sea lanternsharks. New photophore assemblages were described and their arrangement suggests an aposematic use of luminescence to reveal the presence of the dorsal spines. In Etmopteridae, a morphological divergence might be involved in a convergent function, aposematism. Despite the absence of venomous apparatus, the defensive use of spines is documented for the first time by in situ video recordings. Development of highly sensitive underwater video recording devices could allow footage of bioluminescence to be recorded, revealing the use of living light by deep-sea sharks during encounters with conspecifics or predators.

## Additional files

Additional file 1: Animated GIF of MRI transversal section of *Etmopterus spinax* at the level of spine base, going from the tip to the base of the spine. (GIF 557 kb)

Additional file 2: Animated GIF of CT scan sagittal section of *Etmopterus spinax* dorsal spine and fin, starting from the body (1) till the end of the spine (4) and backward. (GIF 74 kb)

Additional file 5: Pictures of injured *E. molleri* collected by deep sea rod fishing (A) Tail cut; (B) ventral side open; (C) closer view of B; (D) another occurrence of ventral bite. Scale bar A = 3 cm, B –C –D = 4 cm. (TIF 19094 kb)
