# Supplementary figures and images for: Etmopteridae bioluminescence: dorsal pattern specificity and aposematic use
Source: Zoological Lett. 2019 Mar 6;5:9. doi: 10.1186/s40851-019-0126-2 (PMC6402137; doi:10.1186/s40851-019-0126-2)

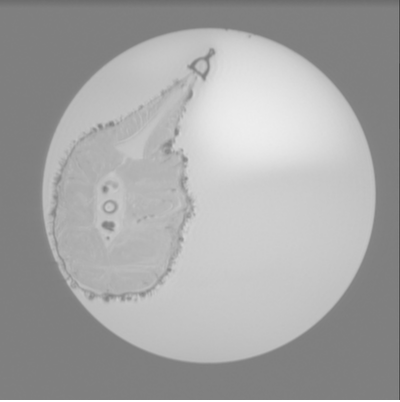

Supplement: Supplementary file 3 — Animated GIF of MRI transversal section of Etmopterus spinax at the level of spine base, going from the tip to the base of the spine. (GIF 557 kb) [file 40851_2019_126_MOESM1_ESM.gif]

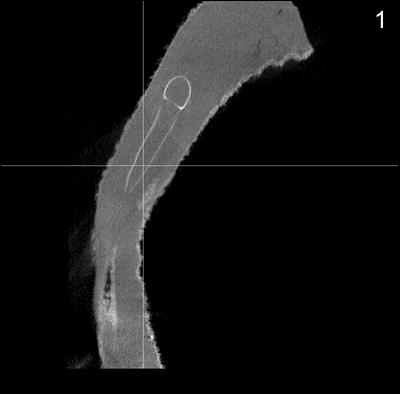

Supplement: Supplementary file 4 — Animated GIF of CT scan sagittal section of Etmopterus spinax dorsal spine and fin, starting from the body (1) till the end of the spine (4) and backward. (GIF 74 kb) [file 40851_2019_126_MOESM2_ESM.gif]

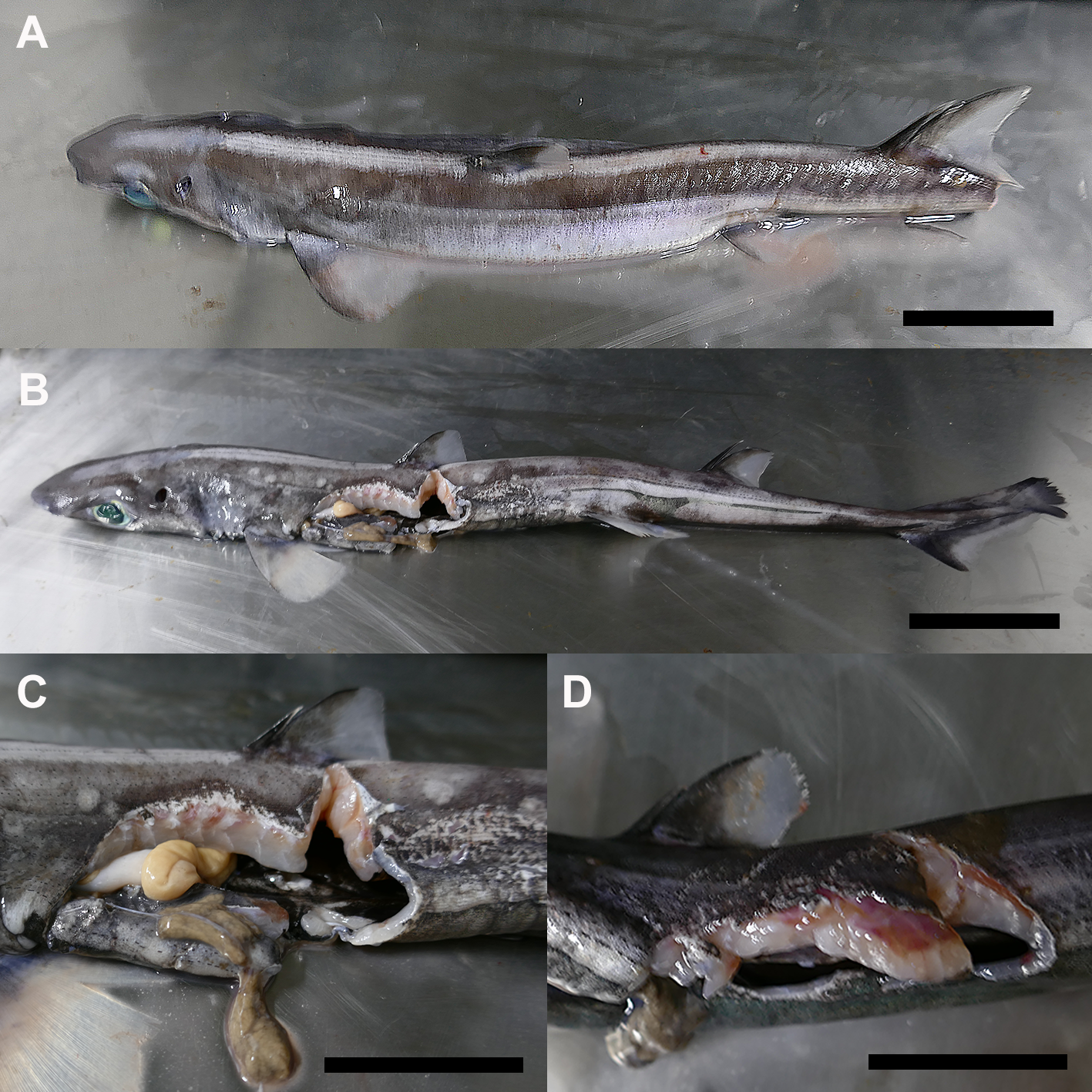

Supplement: Supplementary file 5 — Pictures of injured E. molleri collected by deep sea rod fishing (A) Tail cut; (B) ventral side open; (C) closer view of B; (D) another occurrence of ventral bite. Scale bar A = 3 cm, B –C –D = 4 cm. (TIF 19094 kb) [file 40851_2019_126_MOESM5_ESM.tif]
